# Attention Bias Test Measures Negative But Not Positive Affect in Sheep: A Replication Study

**DOI:** 10.3390/ani10081314

**Published:** 2020-07-30

**Authors:** Jessica E. Monk, Caroline Lee, Emily Dickson, Dana L. M. Campbell

**Affiliations:** 1 Agriculture and Food, Commonwealth Scientific and Industrial Research Organisation (CSIRO), Armidale, NSW 2350, Australia; caroline.lee@csiro.au (C.L.); edickso2@myune.edu.au (E.D.); dana.campbell@csiro.au (D.L.M.C.); 2 School of Environmental and Rural Science, University of New England, Armidale, NSW 2351, Australia

**Keywords:** affective state, behaviour, cognitive bias, emotion, livestock, Merino, welfare

## Abstract

**Simple Summary:**

Attention bias tests may provide a practical measure of emotional states in livestock. An attention bias test has been developed as a measure of negative emotional states in sheep. This study aimed to determine whether the test could also be used to assess positive emotional states. Our results indicated that the attention bias test was unable to differentiate control animals from those in drug-induced positive states (*p* > 0.05). However, our findings further supported the suggestion that the attention bias test may measure negative emotional states in sheep. With further refinement, the attention bias test may be a useful tool to assess and improve livestock welfare.

**Abstract:**

An attention bias test has been developed as a measure of negative affective states in sheep. The test measures an individual’s allocation of attention between a threatening (previous location of a dog) and positive (conspecific photo) stimulus over a 3 min period. This study replicated a previously inconclusive study, to determine whether the test could assess positive affective states under more controlled conditions and with a younger population of animals. Pharmacological treatments were used to induce anxious, calm, happy, and control affective states prior to entering the attention bias test arena (*n* = 20/treatment). We hypothesized that sheep in positive and negative affective states could be differentiated using key measures of attention during testing, including vigilance (head at or above shoulder height) and duration looking towards the valenced stimuli. Anxious sheep were more vigilant than control animals during attention bias testing as predicted (linear mixed effects model, *p* = 0.002), but the positive groups did not differ from controls (*p* > 0.05). There was no effect of treatment on looking behaviors (*p* > 0.05). We suggest this attention bias test paradigm can assess negative but not positive affect in sheep and that modifications to the ethogram or stimuli are needed to more clearly characterize the direction of attention during testing.

## 1. Introduction

As the stakeholders of livestock industries become increasingly concerned about animal welfare, there is a push towards humane production systems that consider the mental well-being of livestock and the promotion of positive welfare states [1,2,3,4]. “Affect” describes an animal’s behavioral, physiological and psychological state, and is thought to vary along the dimensions of valence (pleasant or unpleasant) and arousal (level of activation) [5,6,7]. For the purpose of this study, discrete terms such as “anxious”, “calm”, and “happy” are used to describe an animal’s potential affective state, based on the use of pharmacological manipulations. Importantly though, we consider that animals experience affective states on a continuum along the dimensions of valence and arousal [5,6,7], and use these terms as convenient labels to describe an animal’s expected position along those dimensions. As such, “anxiety” describes a negatively-valenced, high-arousal state, while “calm” and “happy” describe positively-valenced states with low and high degrees of arousal respectively.

There is currently a lack of robust measures of affective state for non-human animals, however one approach which has shown promise is the assessment of affect-driven attention biases [8]. An attention bias is the tendency to process certain types of information before others, which can manifest as increased allocation of attention towards one type of stimulus over another [9]. Biases in attention are determined by the salience of the information presented, or its perceived importance to the individual, and can be influenced by affective state. For example, studies in humans have demonstrated that people in anxious states pay more attention towards threatening stimuli than non-anxious individuals [9,10,11,12] and humans in positive states display attention biases towards rewarding stimuli [13,14]. Methods for assessing attention bias in humans have included eye tracking and looking time tasks that assess gaze fixation on competing images of emotional stimuli [15,16,17]. These methods were first adapted for use in non-human animals by Bethell et al. [18]. The test paradigm has since been adapted for a range of animals species [19], including sheep, showing promise as a rapid and practical measure of anxiety-like states in particular [20].

The attention bias test method for sheep described by Lee et al. [20] in 2016 involved measuring allocation of attention between a threatening stimulus (dog) and a positive stimulus (food). Upon entering the attention bias test arena, sheep could see a dog sitting quietly through a small window for 10 s, then the window was closed, the dog was removed and sheep remained in the test for a further 3 min. Measures of attention were then taken including duration looking towards the closed dog window, vigilance with the head at or above shoulder height and allocation of attention towards the positive stimulus evidenced through latency to feed and duration eating. To determine whether the method could be used to assess affect, pharmacological models of increased and decreased anxiety-like states were used to induce contrasting affective states. The ′anxious′ sheep spent more time looking towards the dog window, spent longer displaying vigilance behavior and took longer to eat compared to the less anxious treatment group. Thus, Lee et al. [20] were able to demonstrate that the test paradigm could measure shifts in attention towards a threatening stimulus and away from a positive stimulus, and that this attention bias was influenced by anxiety-like states in sheep.

Several studies have since been conducted to refine the attention bias test method described by Lee et al. [20] or to validate the effect of different affective states on behavioral responses during testing. Monk et al. [21] refined the method by removing the short habituation period used previously and shortening the duration of exposure to the dog to 3 s. Their study confirmed the previously observed effect of anxiogenic and anxiolytic drugs on behavioral responses during testing. Verbeek et al. [22] used a variation of the method described by Lee et al. [20] and found some unexpected evidence of an attention bias away from the threat in sheep which had been chronically stressed by lying deprivation. This finding was not replicated when sheep were chronically stressed using a pharmacological model, which had no effect on animal behavior during the attention bias test [23]. Each of these attention bias studies used food as a positive stimulus and will broadly be referred to as the original test method hereafter.

To remove the potential influence of appetite on animal responses during testing, Monk et al. [24] changed the positive stimulus to a photograph of a conspecific. During their study, sheep quickly approached and sniffed the novel conspecific photograph and spent a high proportion of time standing next to the photograph, which was interpreted as a positive response to the alternative stimulus. The modified method was then validated using pharmacological models of anxiety-like and depression-like states. The study showed that anxious and depressed groups could be differentiated by their responses in the test, however, the anxious group unexpectedly displayed an attention bias towards the conspecific photograph instead of the threat. It was suggested that anxious sheep displayed greater attention to the social stimulus because flocking behavior is an important response to the threat of predation for sheep [25]. Thus, the photograph was a more salient stimulus than the previous location of the threat. Importantly, the anxious and depressed groups could still be differentiated using the modified paradigm, by considering looking and vigilance behaviors together.

Most recently, Monk et al. [26] aimed to determine whether the modified method could discriminate positive and negative affective states, by using pharmacological models of calm-like, happy-like and anxiety-like states. The results of their study did not support the hypothesis that the modified method could assess positive affect. However, animal responses were potentially confounded by the presence of background noise during testing, the relatively older test animals used in the study or the use of an intramuscular route of administration for the drug diazepam, compared to an intravenous route used previously. Replication of the study conducted by Monk et al. [26] in a quieter environment with a younger population of animals would therefore be useful to determine the extent to which these potentially confounding factors impacted on animal behavior and to more clearly determine whether the modified method can discriminate pharmacologically induced positive and negative affect. Importantly though, animal responses in an attention bias test will need to be replicable across different environments and populations of animals before the method can be applied more broadly as a standardized measure of affect in sheep.

This study aimed to replicate the study conducted by Monk et al. [26] while eliminating any confounding factors that may have impacted on their results, to determine whether the attention bias test can discriminate positive and negative affective states. It was hypothesized that the anxious sheep would be more vigilant than control animals, while the calm and happy groups were expected to be less vigilant. The positive treatment groups were expected to spend more time looking towards the conspecific photo and less towards the dog window relative to control animals. However, in opposition to human studies, we expected the anxious group would also show increased attention towards the conspecific photo and less towards the threat. This expectation was due to the salience of the social stimulus for the sheep as a flocking species, and is consistent with the findings of Monk et al. [24] and tendencies observed by Monk et al. [26]. We had no a priori predictions for the differences in looking behaviors between the anxious and positive groups. Body temperature responses were recorded to assess the physiological impacts of drug treatments and attention bias testing. Injection with mCPP was expected to cause stress-induced hyperthermia [27,28], while diazepam was expected to attenuate internal body temperature response to attention bias testing, consistent with an anxiolytic effect [27,29]. Morphine was not expected to impact on body temperature at the dosage given, consistent with the previous study [26].

## 2. Materials and Methods

### 2.1. Ethical Statement

The protocol and conduct of the experiment were approved in October 2018 by the CSIRO F.D. McMaster Laboratory Animal Ethics Committee (AEC19-15), under the New South Wales Animal Research Act 1985. All sheep were monitored closely during and after the experiment for adverse responses to the drug treatments and test protocol.

### 2.2. Animal Details

The experiment was conducted in Armidale, NSW, Australia over a duration of 2 days in October 2019. In total, 80 one-year-old maiden Merino ewes with an average bodyweight of 54.8 ± 6.8 kg were used. Sheep were kept at pasture with ad-libitum access to water before and after the testing periods. Sheep had been bred, raised and tested on the same experimental farm. Sheep had been managed extensively and had prior experience with dogs on-farm during routine management and movement between handling facilities, but did not have experience with the attention bias test.

### 2.3. Experimental Design

The current study replicated the protocol described by Monk et al. [26] with a few key changes. First, animals used in the current study were one-year-old female sheep, as opposed to 7-year-old female sheep used previously. Second, the drug diazepam was administered intra-venously in the current study while the previous study administered diazepam intra-muscularly at the same dose rate. Finally, the conditions on the experimental farm at the time of the study were carefully controlled such that background noise was minimal, limiting potential interference with animal behavior during testing. All other aspects of the methodology remained the same as described by Monk et al. [26].

Readers are directed to Monk et al. [26] for a detailed description of the test methods and pharmacological models. Briefly, 80 sheep were randomly allocated to one of four treatment groups balancing for bodyweight: Anxious, Control, Calm, or Happy (*n* = 20 per treatment). Drugs were used to pharmacologically induce contrasting affective states in each group prior to testing in an attention bias test [20,24]. The Anxious group received an intramuscular (i.m.) injection of m-chlorophenylpiperazine (mCPP; Tocris, Bristol, UK) at a dose rate of 1.5 mg/kg to induce an anxiety-like state [30,31]. Control animals received an i.m. injection of 1.5 ml of BP saline (Baxter, Old Toongabbie, Australia). The Calm group received an intra-venous injection of diazepam (Troy Laboratories, Sydney, Australia) at a dose rate of 0.1 mg/kg to induce a calm-like state [30,32]. The Happy group received an i.m. injection of morphine (Hospira, Melbourne, Australia) at a dose rate of 1 mg/kg to induce a euphoric or happy-like state [33,34]. Intra-venous injections were administered into the jugular vein while i.m. injections were administered into the rump. All injections were administered 30 min prior to testing in the attention bias test. Sheep were divided into two cohorts (*n* = 40) balancing for treatment group, to be tested on separate days for logistical reasons.

### 2.4. Attention Bias Test Method

**Figure 1:**
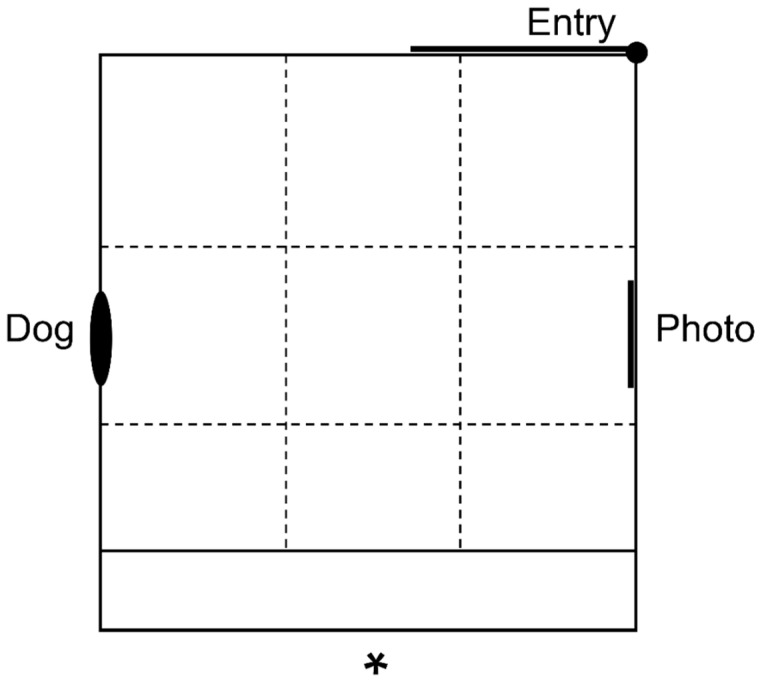
Diagram of the attention bias test arena. A dog was visible through a small window for 3 s at the beginning of the test, then an opaque covering was lowered over the window and the dog was removed. ‘*’ denotes the position of a camera and hidden observer, located on the second story of an adjacent building. External walls of the arena were covered in opaque matting 1.8 m high. A small section within the arena was fenced off to prevent sheep moving into the corners out of view of the camera. The accessible area of the arena was 4 × 4.2 m. The dashed lines represent the position of a 3 × 3 grid overlaid on video footage for calculation of zone related behaviors.

The attention bias test arena consisted of a 4 × 4.2 m area with 1.8 m high opaque walls and a concrete floor (Figure 1). A photograph of the arena is shown in Monk et al. [26]. An approximately life-size photograph of an unfamiliar Merino sheep was positioned on one wall of the arena. A high-quality copy of the photograph was made available for download by Monk et al. [24]. On the wall opposite the conspecific photo was a small window, behind which a dog sat or stood quietly. The dog was an approximately 10-year-old male border collie and was the same dog that was used in Monk et al. [26] and Monk et al. [24]. The dog was visible at the beginning of the test when an individual sheep entered the arena through a gate. A timer began once the gate was closed and the sheep had made visual contact with the dog, evidenced by the test animal orienting its head directly towards the dog and exhibiting a freezing response. The dog remained visible for 3 s, then an opaque cover was lowered over the window, the dog was removed and the sheep remained in the arena for 3 min while behavioral observations were made (Table 1). A video is provided in the Supplementary Materials to demonstrate how the beginning of the test is conducted and to give examples of key behaviors recorded during testing (Supplementary Video S1). Sheep were also monitored for abnormal behaviors, including shaking of the tail, head, or body, ataxic gait, and head rolling, which have been observed previously in sheep treated with mCPP [31].

### 2.5. Body Temperature

Internal body temperatures were recorded prior to, during and after attention bias testing using Thermochron iButtons^®^ (Model number DS1922L-F5, sample rate 1 min; Embedded Data Systems, Lawrenceburg, IN, USA). iButtons were attached to progesterone-free Controlled Internal Drug Release devices (CIDR^®^; Zoetis, Melbourne, Australia) and were inserted into the vagina of each sheep one day before testing [35]. Temperature data were extracted at times −30, −20, −10, −1, 6, 11, 16, 21, and 26 min relative to the beginning of attention bias testing, using eTemperature version 8.32 (OnSolution, Castle Hill, Australia). These times best represented the average baseline and peak temperature responses to testing, based on preliminary visual observation of the data. Data from 5 loggers were missing due to technical faults. The first 5 animals tested on day 1 were missing some temperature data between times −30 and 0 due to technical faults.

### 2.6. Statistical Methods

Data were analysed using R version 3.6.0 [36], as described by Monk et al. [26]. *p* values less than 0.05 were considered significant. The normality and homoscedasticity of model residuals were confirmed using Shapiro–Wilks test for normality and the visual assessment of Q-Q and residuals vs. fitted values plots. All mixed models fitted treatment as a fixed effect and cohort (test day) as a random effect. A Tukey method of *p*-value adjustment was applied for post-hoc multiple comparisons. Effect sizes were estimated for some differences between groups using Pearson′s correlation coefficient *r* [37].

Linear mixed effects models were used to analyze attention data, vigilance duration, and duration standing near the conspecific photo using the package nlme [38]. Attention to photo and vigilance duration data failed to meet parametric model assumptions for normality of residuals and were log transformed. The transformed vigilance duration data also failed to meet parametric model assumptions and were analysed using a Kruskal–Wallis non-parametric ANOVA. Post-hoc multiple comparisons for the Kruskal–Wallis ANOVA were performed using the package pgirmess [39]. Both the parametric and non-parametric model outputs for vigilance duration data have been reported.

Generalized linear mixed effects models were used to model count data using the package lme4 [40]. Photo sniff frequencies and the number of zones entered were fitted with a Poisson distribution and log link function. Environment sniff frequency and zones crossed were fitted with a negative binomial distribution and log link function, due to evidence of over-dispersion in the data. Vocalization data were analysed with negative binomial hurdle models to account for excess zeros in the data, using the package pscl [41]. Fisher′s exact tests were used to analyze the number of animals in each group that entered the zone closest to the dog window, sniffed the closed window, urinated and performed abnormal head and tail shaking behaviors. Post hoc multiple comparisons were made using the package rcompanion [42].

Latencies to become non-vigilant, sniff the photo and sniff the environment were analysed using Cox’s proportional hazards models, fitting treatment as a fixed effect [21,43,44]. Latencies were deemed as censored results if animals failed to perform the given behavior within 180 s.

Body temperature data were analysed using a linear mixed effects model, fitting fixed effects of treatment, time and a treatment × time interaction. To account for repeated measurements on individuals over time, sheep identity nested within time was fitted as a random effect in the model.

## 3. Results

### 3.1. Vigilance and Attention

**Figure 2:**
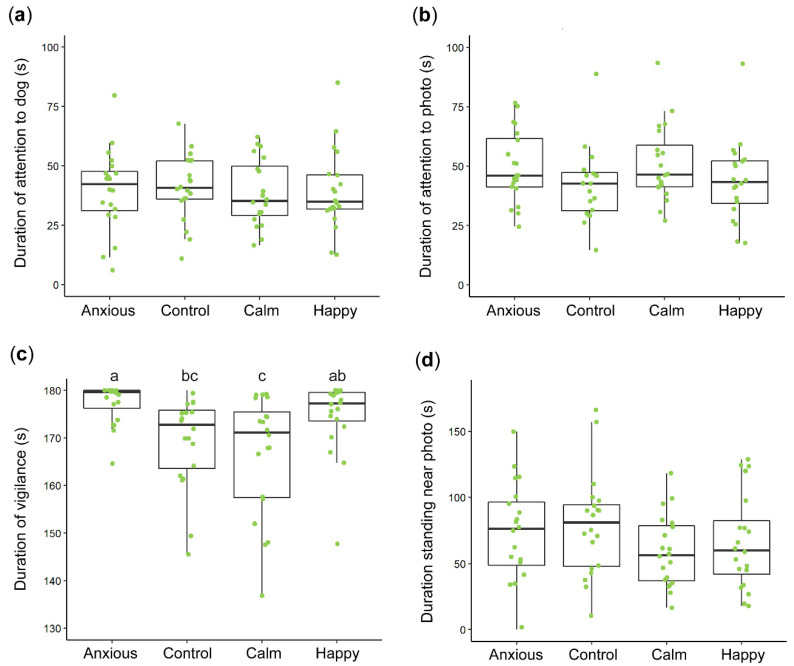
Boxplots summarizing the raw data within each treatment group for looking behaviors (**a**,**b**), vigilance (**c**) and duration spent standing near the photo (**d**). Boxplots display the median durations, the interquartile range (IQR) and the range of data within 1.5× the IQR. The raw duration data for individuals within each treatment group are represented by the dots. Data were analyzed using linear mixed effects models. Significant differences between groups (**a**–**c**) were only found for vigilance data. Note that the plot axes are scaled differently.

**Figure 3:**
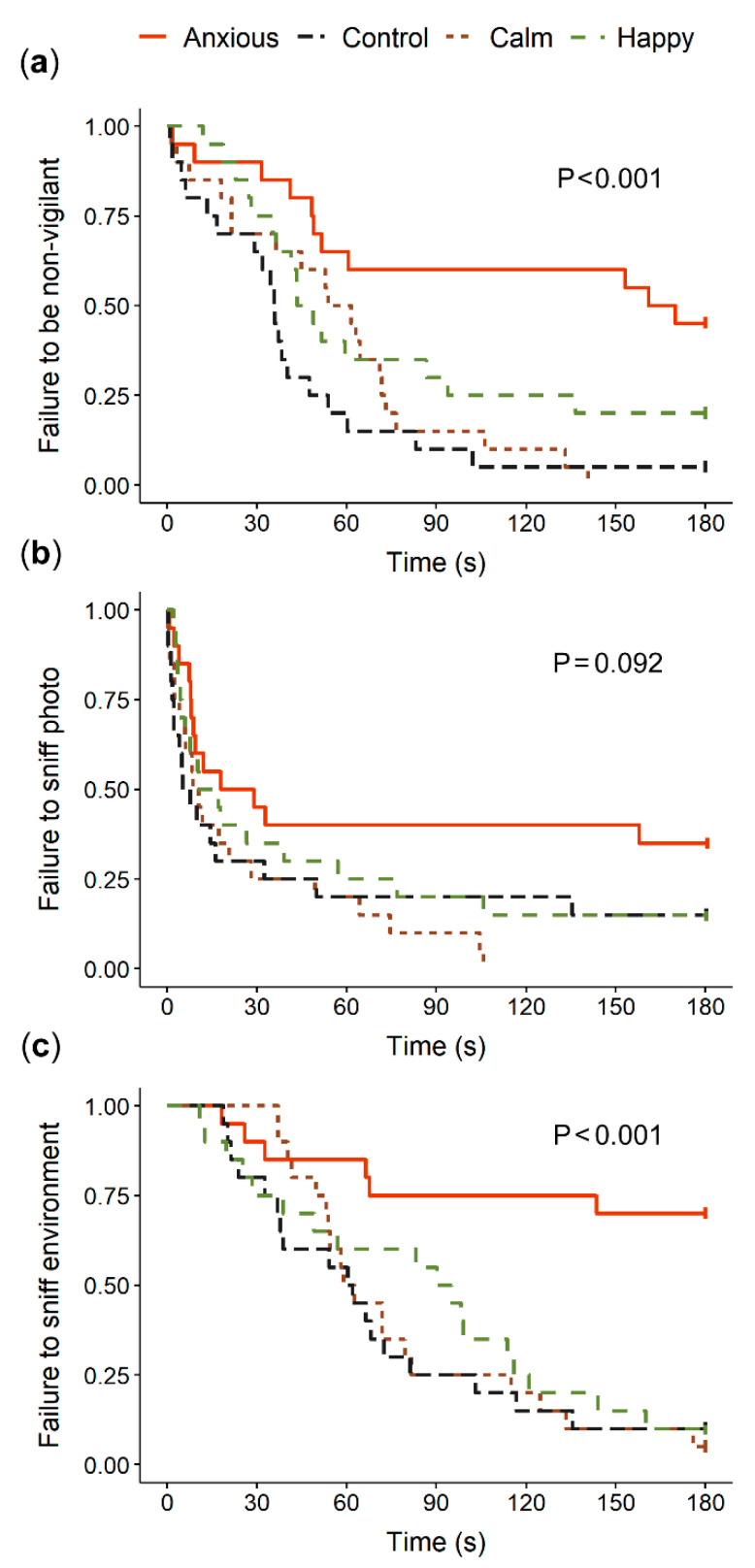
Kaplan-Meier curves for latency to become non-vigilant (**a**), sniff the photo (**b**) and sniff the environment (**c**). The probability on the *Y*-axis drops at each time the given behavior was exhibited by an individual in the treatment group.

Figure 2 summarizes the raw vigilance and looking data. Anxious sheep spent significantly more time displaying vigilance behavior than Control animals (t (75) = −2.7, *p* = 0.009, r = 0.30) while the positive treatment groups did not differ from Controls (Table 2). Anxious sheep took significantly longer to become non-vigilant than all other groups (Table 3, Figure 3). Latency to become non-vigilant for the Happy group was intermediate between the Control and Anxious groups, while the Calm group did not differ from Controls (Table 3, Figure 3). Linear mixed effects models showed no differences between treatment groups for duration looking towards the dog window (F (3,75) = 0.1, *p* = 0.967) or photo (F (3,75) = 2.0, *p* = 0.141; Figure 2).

### 3.2. Other Behaviors

The Anxious treatment group sniffed the photo and environment significantly less than all other groups, while the other treatment groups did not differ from one another (Table 2). The Anxious sheep displayed a longer latency to sniff the environment and tended to display a longer latency to sniff the photo than Control animals (Table 3, Figure 3). Only four animals in total sniffed the closed dog window (Table 2).

Figure 2 summarizes the raw data for duration standing in the zone closest to the photo. There were no differences between groups for duration standing near the photo (Table 2). The positive treatment groups crossed significantly more zones than the Anxious group, but no treatments significantly differed from Controls (Table 2). The effects of treatment on zones entered and number of animals which entered the zone closest to the dog window were not significant (Table 2).

The effects of treatment on open- and close-mouthed bleats were not significant (Table 2). More sheep in the Anxious group urinated during testing than the other groups (Table 2).

No animals showed signs of ataxic gait or performed body shaking and head rolling behaviors. Ten sheep showed tail shaking and nine sheep showed head shaking behaviors. These behaviors occurred more in the Anxious group but were not limited to the Anxious group (Table 2). It was unclear from video footage whether head and tail shaking behaviors occurred due to the presence of flies or as an abnormal response to the drugs.

### 3.3. Body Temperature

**Figure 4:**
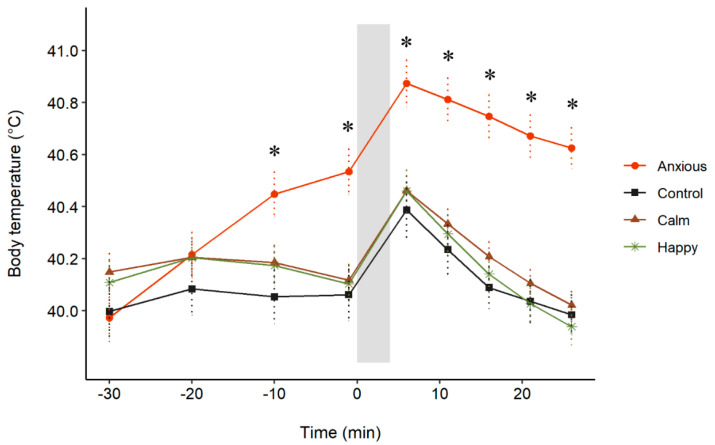
Mean s.e.m. body temperature for each treatment group before and after attention bias testing. All drugs were administered at time −30. Attention bias testing occurred between times 0 and 3 (grey bar). The “*” symbols denote a significant difference between the Anxious group and other treatment groups at a given time point, determined using a repeated measures linear mixed model. No differences were observed between the Control, Calm and Happy groups.

The repeated measures analysis on body temperature data showed a significant Treatment × Time interaction (F (24,557) = 18, *p* < 0.001; Figure 4). Contrasts indicated that body temperatures did not differ between any of the groups at times −30 or −20 (*p* > 0.1). The Anxious group had a significantly higher body temperature than all other groups at all other time points (*p* < 0.01, *r* = 0.27−0.57). No differences in temperature were observed between the Control, Calm, and Happy groups.

## 4. Discussion

Monk et al. [26] suggested that background noise, animal age, or changes to the route of administration of pharmacological agents may have confounded behavioral responses during attention bias testing in their study. The current replication study showed few changes to the results after removing these potentially confounding effects, suggesting that other factors played a more important role in determining animal responses during testing.

In contrast with our hypotheses, the positive treatment groups did not differ from Control animals for vigilance behavior in either the current or previous study [26]. This may indicate that the modified attention bias test method cannot be used to measure positive affect. The test environment itself could bring about a negative emotional state, as it involves novelty and isolation that can both be very stressful for sheep [45,46]. This is particularly true for the highly gregarious Merino breed [46,47], which was used in the current and previous studies. Thus, the current test paradigm may override the effects of drug treatments or environmental manipulations that induce a positive affective state prior to testing. Alternatively, test animals could have been in a positive affective state prior to testing, such that the pharmacological manipulation did not strongly improve affect relative to the Control group. However, due to the stressful nature of handling and novel environments for sheep we suggest it is unlikely the sheep were in a positive state prior to testing. Further studies are required to determine whether the original attention bias test methodology, which used food as a positive stimulus, can discriminate positive affective states. However, it may be the case that an anxiety-inducing test paradigm such as this cannot effectively measure positive affect in gregarious species such as the sheep.

Although we suggest that the affective states of sheep treated with diazepam or morphine may have been negatively impacted by the anxiety-inducing attention bias test, we might still expect these sheep to be in a less negative state than Control animals. Consequently, these treatment groups were still expected to display reduced vigilance relative to Control animals as observed previously for diazepam-treated sheep [20,21], however this was not the case. One important factor which could limit ability to detect less negative affective states using the modified methodology in particular, is that sheep are not given a strong incentive to become non-vigilant. In previous studies using the original method, vigilance and attention towards the positive stimulus, evidenced through feeding behaviors, were mutually exclusive and represented a clear shift in the underlying systems driving behavioral responses from defense motivation to hunger motivation [48]. In the current study, sheep could still display attention towards the positive stimulus while maintaining a vigilant head position. Without a strong incentive to become non-vigilant, it may be difficult to differentiate those sheep which would be willing to compromise vigilance but do not have any incentive to do so, from those which are not willing to compromise vigilance. Further, in the absence of any rewards, positive affect is known to broaden attention to the external environment [49], which also could have resulted in an increased observation of vigilance in positive groups compared to our initial expectations.

In addition to preventing the assessment of less negative states, the tendency for mean duration spent expressing vigilance behavior to approach the maximum test duration reduces the sensitivity of the test to negative affective states as more data became censored at 180 s. Duration of vigilance in the Anxious group was higher than the Control group across both the current and previous studies, however this difference was only statistically significant in the current study. Importantly though, the mean durations reported by Monk et al. [26] were comparable to the current study. Back-transformed estimated mean durations of vigilance for the Anxious group during the previous and current studies were 176 and 177 s respectively, while the Control group means were 170 and 170 s respectively. Thus, the statistically significant difference reported in the current study was not necessarily due to decreased vigilance in Control animals, but was more likely due to changes in the amount of variation present within the treatment groups. Such a change in variation is apparent when comparing the boxplots presented in each article, summarizing the raw data [26] (Figure 2 in both articles). Studies using feed as a positive stimulus have shown lower mean durations of vigilance in Control animals, with reported values ranging from 123 s to 165 s [20,21,23], excepting a high duration of 176 s reported by Verbeek et al. [22]. Thus, the original test method, in which sheep are motivated to become non-vigilant to feed, may be better able to assess affect using vigilance behavior.

Duration of looking towards the dog window and conspecific photograph did not differ between any treatment groups in our study, which contrasts with our hypotheses but is consistent with the findings of Monk et al. [26]. One important factor that may limit the interpretation of looking behavior as a key measure of attention during testing is the use of a threatening stimulus that does not remain visually present for the entire test duration. The presence of a live dog during behavioral tests is known to be highly aversive for sheep, reducing and even eliminating the occurrence of exploratory behaviors in other contexts [50,51]. Within the attention bias test, removal of the dog after 3 s effectively reduces the intensity of the threat, allowing sheep to display a greater range of behaviors that are not specifically focused on predator avoidance. However, by removing the visual component of a threatening stimulus, sheep can no longer localize their gaze towards the threatening stimulus itself, only to the last known location of the threat. While this approach has proven successful in some attention bias studies [20,24], differences in looking behaviors between control and treated animals have not been consistent in all studies [21,26]. The use of a negative stimulus that remains present for the entire test duration, as is the case for the positive stimulus, may allow a clearer and more consistent characterization of visual attention in future studies.

Another important factor to consider is that head orientation alone may not effectively characterize visual attention in a species with a wide field of vision, such as the sheep [52,53]. Duration of looking with binocular vision has been considered as a key measure of attention in each version of the attention bias test. Head orientation has also been used to define visual attention in other studies using sheep [54,55] and goats [56]. This is because sheep will typically orient their head towards a point of interest, such as a predator threat, so that it is situated within their narrow field of binocular vision, allowing for depth perception and improved visual acuity [53]. However, this definition of attention does not account for attention directed towards other parts of the visual field. Further, when confronted with a known predator threat with which sheep no longer have visual contact, greater attention to movement within the peripheral vision and to other senses such as hearing or olfaction may be more beneficial to the survival of the sheep. Thus, there may be a need to reevaluate this definition of attention, particularly in a context where the threat is not visually present.

Within the current test paradigm, sniffing behaviors may indicate olfactory attention, and have been shown to differ significantly between Anxious sheep and other treatment groups in the current and previous studies [24,26]. Auditory attention can be characterized during testing by assessing ear postures [57,58], however, assessment of ear postures is labor-intensive and time-consuming and will limit the practical application of the method. Further, we suggest that the assessment of auditory and olfactory attention will be more meaningful in test paradigms that incorporate auditory and olfactory cues associated with the positive and negative stimuli. Indeed, the potential to use auditory stimuli to assess attention bias in sheep has already been explored with promising results, using behavior to assess lateral attention towards audio stimuli that were simultaneously played on either side of the animal [58]. It should also be considered that the stimuli presented in this study were cross-modal. A live dog can be perceived through more sensory modalities than the conspecific photograph, which is attended to through vision but does not have a scent, sound, or tactile feel that is consistent with the visual representation of the conspecific. Within the test paradigm developed by Lee et al. [20], inclusion of balanced stimuli that can be perceived through a number of sensory modalities and modification of the ethogram accordingly may strengthen the characterization of attention, and consequently attention biases, during testing.

An alternative explanation for not seeing any clear effects of treatments on behavior during testing in the current study could be that the pharmacological treatments did not induce the expected affective states. In previous attention bias studies, diazepam treated sheep showed decreased vigilance behavior relative to other treatment groups, suggesting these animals were in a calmer affective state. However the differences between diazepam treated sheep and control animals were not statistically significant in either study [20,21]. It was also suggested that sheep may have spent more time eating and consequently less time displaying vigilance behavior due to diazepam increasing appetite and not due to an anxiolytic effect of the drug [59,60]. Further, studies in rats have found that diazepam can impair spatial memory or learning [61,62], which may confound responses during attention bias testing if a sheep no longer associates the previous location of the dog with the threat of a dog and does not localize their attention accordingly. Previous studies administering diazepam to sheep have given inconsistent results, with some [32] but not all studies [30,31] observing behavioral responses that are expected to indicate a calm state, such as decreased vigilance during an arena test, decreased agitation in an isolation box and a reduced startle response. Further, we had expected diazepam treated animals to show an attenuated internal body temperature response to testing which was not the case, and which was also not observed in the previous study [26]. Together these findings suggest that diazepam may not have been an effective model for calm affective states at the dose rate used in this study.

Morphine has only been used as a model of positive affect in sheep in a limited number of studies and may not be producing the desired effects [26,33,63]. Previously, it was suggested that morphine primarily acts on the arousal component of affect without a clear effect on the valence component [26]. In the current study, morphine treated sheep displayed more open-mouthed bleats than other groups, but the difference was not statistically significant. They also did not show a significant increase in locomotor activity during testing. Thus, in contrast to previous studies, the potential impact of morphine on arousal was also less clear in this study. Considering the inconsistencies between studies using both diazepam and morphine, it is suggested that further work is needed to confirm the effects of these drugs as pharmacological models of positive affect in sheep, and to explore alternative pharmacological and environmental models.

Sheep treated with the drug mCPP have displayed behaviors associated with anxious states with reasonable consistency across the studies in which it was used. In the current study, the Anxious group showed increased vigilance as expected and a clear body temperature response indicating stress-induced hyperthermia [29]. They were also less likely to sniff the environment or photograph and crossed the fewest zones, suggesting a general unwillingness to explore the novel environment which is associated with fearfulness and anxiety [64,65]. Nevertheless, the continued observation in the current and previous studies of unwanted side-effects in sheep treated with mCPP at a lowered dose rate of 1.5 mg/kg remains problematic [26]. Further, its potential impact on appetite means it may not be suitable for use in studies where feed is presented [66], although it should be noted that a reduction of appetite may be a function of increased anxiety or stress and not an independent effect [67,68]. Overall, we suggest mCPP likely had the desired effect on anxiety in this study, but that further studies should explore alternative pharmacological or environmental models of anxiety-like states for sheep, including commercially relevant housing or husbandry changes that are likely to impact on affect. Further, models that target a range of physiological pathways or mechanisms may be useful to confirm that the responses observed during testing are due to negative affective states more generally and are not specifically tied to the serotonergic pathway primarily targeted by mCPP [69].

## 5. Conclusions

The attention bias test paradigm developed by Lee et al. [20] has shown promise as a measure of negative affective state in sheep. However, the modified test used in this study was not able to discriminate Control animals from sheep in a pharmacologically-induced positive affective state. It is suggested that an inherently aversive test paradigm such as this attention bias test, which involves novelty and isolation, may not allow for the assessment of positive affect in a gregarious species such as the sheep. The modified method, which uses a conspecific photograph instead of food as a positive stimulus, may not provide sheep with a strong enough incentive to become non-vigilant during the test, limiting its ability to discriminate affective states. It is therefore recommended that further studies utilize the attention bias test method described by Monk et al. [21] using food as a positive stimulus, or explore alternative stimuli for use in the test. Modifications to the stimuli and ethogram may allow for a clearer characterization of attention biases during testing. The pharmacological models used across the attention bias studies require further validation to ascertain their influence on affect in sheep and other non-human animals.

## Figures and Tables

**Table 1:** Ethogram of behaviors recorded during the attention bias test [24].

Behavior	Definition
Attention to dog	Duration spent looking at the dog window wall with binocular vision (*s*), where direction of binocular vision was assessed using head orientation, observed from video footage taken at a top-down angle (Video S1)
Attention to photo	Duration spent looking at the conspecific photo wall with binocular vision (*s*)
Vigilance	Duration spent with the head at or above shoulder height (*s*) and latency to become non-vigilant (*s*)
Sniff photo	Frequency of sniffing (*n*) and latency to sniff the conspecific photo (*s*)
Sniff environment	Frequency of sniffing (*n*) and latency to sniff the floor or walls of the arena (*s*)
Zones crossed	Number of zones crossed in the arena with two front feet in a new zone or one front foot in the new zone and the other on the line (*n*)
Zones entered	Number of available zones entered (*n*; ranging from one to nine zones available within the arena, each new zone entered was only counted once during the test)
Zone durations	Duration spent standing in the zone closest to the conspecific photo (*s*) and the number of animals that entered the zone closest to the dog window (*n*; analysed instead of duration due to low number of entries into zone)
Vocalizations	Number of open-and close-mouthed bleats during testing (*n*)
Urinations	Number of urinations (*n*)

**Table 2:** Mean ± s.e.m. behavioral responses of sheep during the attention bias test.

Behavior	Anxious	Control	Calm	Happy	Statistical Test	Test Value (df)	*p*-Value
Vigilance duration (s) ^1,2^	5.12 ± 0.01 (177) ^a^	5.13 ± 0.01 (169) ^bc^	5.11 ± 0.01 (166) ^c^	5.16 ± 0.01 (174) ^ab^	LME	*F*_(3,75)_ = 6.0	**0.002**
Vigilance (*mean rank*)	56.5 ± 4.5 (177) ^a^	30.9 ± 4.4 (170) ^b^	27.9 ± 4.5 (167) ^b^	46.8 ± 4.8 (175) ^ab^	Kruskal-Wallis	*X*^2^_(3)_ = 20	**<0.001**
Looking towards dog (s)	39.8 ± 3.7	40.9 ± 3.7	39.5 ± 3.7	39.3 ± 3.7	LME	*F*_(3,75)_ = 0.1	0.967
Looking towards photo (s) ^1^	3.9 ± 0.1 (47.5)	3.7 ± 0.1 (39.5)	3.9 ± 0.1 (48.9)	3.7 ± 0.1 (40.7)	LME	*F*_(3,75)_ = 2.0	0.141
Sniff photo (*n*) ^1^	0.4 ± 0.2 (1.5) ^a^	1.3 ± 0.1 (3.5) ^b^	1.4 ± 0.1 (4.2) ^b^	1.0 ± 0.1 (2.8) ^b^	GLME_P	*X*^2^_(3)_ = 25.9	**<0.001**
Sniff environment (*n*) ^1^	−0.4 ± 0.3 (0.7) ^a^	1.4 ± 0.2 (4.0) ^b^	1.3 ± 0.2 (3.8) ^b^	1.1 ± 0.2 (3.1) ^b^	GLME_NB	*X*^2^_(3)_ = 26.7	**<0.001**
Sniff closed window (*n*) ^3^	0	1	2	1	FET	*n*/A	0.900
Standing near photo (s)	72.0 ± 8.0	79.2 ± 8.0	59.1 ± 8.0	66.5 ± 8.0	LME	*F*_(3,75)_ = 1.2	0.336
Zones crossed (*n*) ^1^	2.81 ± 0.1 (16.6) ^a^	2.98 ± 0.1 (19.8) ^ab^	3.35 ± 0.1 (28.4) ^b^	3.25 ± 0.1 (25.9) ^ab^	GLME_NB	*X*^2^_(3)_ = 10.6	**0.014**
Zones entered (*n*) ^1^	1.73 ± 0.1 (5.65)	1.82 ± 0.1 (6.15)	1.97 ± 0.1 (7.20)	1.82 ± 0.1 (6.20)	GLME_P	*X*^2^_(3)_ = 4	0.260
Enter zone close to dog (*n*) ^3^	5	7	13	9	FET	*n*/A	0.070
Open-mouthed bleats (*n*)	9.0 ± 2.3	11.1 ± 2.0	12.2 ± 2.3	25.9 ± 3.6	GLME_NB_H	*X*^2^_(3)_ = 5.5	0.140
Close-mouthed bleats (*n*)	6.7 ± 0.5	9.9 ± 0.7	9.5 ± 0.7	9.6 ± 0.7	GLME_NB_H	*X*^2^_(3)_ = 1.9	0.580
Urinations (*n*) ^3^	7 ^a^	1 ^ab^	2 ^ab^	0 ^b^	FET	*n*/A	**0.008**
Head shaking (*n*) ^3^	5	1	1	2	FET	*n*/A	0.09
Tail shaking (*n*) ^3^	9 ^a^	0 ^b^	0 ^b^	1 ^a^	FET	*n*/A	**<0.001**

^a,b,c^ Significant differences between treatment groups within each row of the table as determined using post-hoc analyses, bold font emphasizes significant *p* values. ^1^ Back-transformed means are reported in parentheses, least squares means are reported on the log scale. ^2^ Note that vigilance durations were censored at 180 s. ^3^ Raw number of animals in each group that exhibited the given behavior are reported. LME; linear mixed effects model fitting cohort as a random effect, GLME; generalized linear model with a Poisson (P) or Negative Binomial (NB) distribution, data with excess zeros fitted hurdle models (H), FET; Fisher′s Exact Test, test statistic is not applicable (*n*/A).

**Table 3:** Output of the Cox proportional-hazards models for latency to become non-vigilant, sniff the photo and sniff the environment.

Latency to	Group	Mean (s) ^1^	Censored (*n*) ^2^	Coefficient ^3^	SE (Coeff)	Hazard Ratio (95% CI)	Wald (z)	*p*-Value	Likelihood Ratio	df	*p*-Value
Non-vigilance	Control	42.7 ^a^	1	Reference					18.2	3	**<0.001**
	Anxious	119.9 ^b^	9	−1.533	0.392	0.22 (0.10–0.47)	−3.91	**<0.001**			
	Calm	56.2 ^ac^	0	−0.345	0.324	0.71 (0.38–1.34)	−1.06	0.288			
	Happy	75.3 ^c^	4	−0.716	0.343	0.49 (0.25–0.96)	−2.08	**0.04**			
	Anxious			Reference							
	Calm			1.188	0.390	3.28 (1.53–7.04)	3.05	**0.002**			
	Happy			0.817	0.395	2.26 (1.04–4.91)	2.07	**0.039**			
	Calm			Reference							
	Happy			−0.371	0.34	0.69 (0.35–1.35)	−1.09	0.277			
Sniff photo	Control	41.5	3	Reference					6.7	3	0.08
	Anxious	77.9	7	−0.713	0.37	0.49 (0.23–1.01)	−1.93	0.053			
	Calm	26.4	0	0.144	0.33	1.16 (0.60–2.20)	0.43	0.665			
	Happy	46.8	3	−0.228	0.33	0.80 (0.41–1.56)	−0.66	0.507			
	Anxious			Reference							
	Calm			0.850	0.36	2.36 (1.16–4.79)	2.37	**0.018**			
	Happy			0.490	0.37	1.63 (0.79–3.36)	1.31	0.190			
	Calm			Reference							
	Happy			−0.372	0.33	0.69 (0.36–1.32)	−1.12	0.262			
Sniff environment	Control	70.5 ^a^	2	Reference					25.8	3	**<0.001**
	Anxious	143.7 ^b^	14	−1.905	0.48	0.15 (0.06–0.38)	–3.97	**<0.001**			
	Calm	79.1 ^a^	1	−0.073	0.33	0.93 (0.49–1.77)	–0.22	0.820			
	Happy	86.1 ^a^	2	−0.262	0.34	0.77 (0.40–1.48)	–0.78	0.430			
	Anxious			Reference							
	Calm			1.832	0.48	6.25 (2.45–15.9)	3.83	**<0.001**			
	Happy			1.643	0.48	5.17 (2.03–13.2)	3.44	**<0.001**			
	Calm			Reference							
	Happy			−0.189	0.33	0.83 (0.43–1.59)	−0.57	0.568			

^1^ Raw mean latencies are reported, significant differences between groups are indicated by superscripts ^a,b,c^, bold font is used to emphasize significant *p* values (<0.05). ^2^ The number of animals in each group that failed to exhibit the given behavior within 180 s. ^3^ Regression coefficients from the Cox proportional-hazards models.

## Data Availability

The dataset generated during this study is publicly available in the CSIRO Data Access Portal (DAP) [Permalink: https://doi.org/10.25919/5ee81fc454dbe].

## Supplementary Materials

The following are available online at https://www.mdpi.com/2076-2615/10/8/1314/s1, Video S1: Conduct of the modified attention bias test and examples of recorded behaviors.
